# Cognitive Decline in Patients with Alzheimer’s Disease and Its Related Factors in a Memory Clinic Setting, Shanghai, China

**DOI:** 10.1371/journal.pone.0095755

**Published:** 2014-04-21

**Authors:** Qianhua Zhao, Bin Zhou, Ding Ding, Satoshi Teramukai, Qihao Guo, Masanori Fukushima, Zhen Hong

**Affiliations:** 1 Department of Neurology, Huashan Hospital, Fudan University, Shanghai, China; 2 Translational Research Informatics Center, Foundation for Biomedical Research and Innovation, Kobe, Japan; 3 Institute of Neurology, Huashan Hospital, Fudan University, Shanghai, China; 4 Department of Clinical Trial Design and Management, Translational Research Center Kyoto University Hospital, Kyoto, Japan; Nathan Kline Institute and New York University School of Medicine, United States of America

## Abstract

**Objectives:**

Progressive cognitive decline is a characteristic hallmark of AD. It is important to identify prognostic markers to improve patient care and long-term planning. We aimed to identify the characteristics of disease progression in AD patients, focusing on cognitive decline and its related factors.

**Methods:**

Clinically diagnosed AD patients in a memory clinic were followed. The mini–mental state examination (MMSE) and a battery of other neuropsychological tests were performed to assess the rate of cognitive decline and to analyze the related factors.

**Results:**

A total of 165 AD patients were analyzed for cognitive changes. The MMSE scores declined at a rate of 1.52 points per year. Most neuropsychological test scores deteriorated significantly over time. Younger and early-onset AD patients deteriorated more rapidly than older and late-onset patients in global cognition and executive function. Men declined faster in memory but slower in attention than women. Higher education was associated with more rapid deterioration in visuo-spatial ability. Family history, hypertension and cerebral vascular disease were also associated with disease progression.

**Conclusion:**

Attention, executive and visuo-spatial functions deteriorate at faster rates than other cognitive functions in AD patients. Age and age at onset were the main factors that associated with deterioration.

## Introduction

As estimated 24 million people worldwide suffer from dementia, the majority of who are thought to have Alzheimer’s disease (AD). The high prevalence of dementia associated with aging, together with the lack of effective therapy, are important global issues. According to an evidence-based Delphi consensus study on dementia, the countries or regions with the largest numbers of affected individuals over the coming three decades are likely to be China and the developing West Pacific countries [1]. The number of people with dementia in China was 9.19 million in 2010, among which AD is 5.69 million [2]. This represents a major public health concern and has been identified as the research priority [3].

Different rates of progression had been observed among patients with AD. Progressive forms of AD have been reported with rapid cognitive decline. And disease duration is only a few years [4]. Improving the estimation of disease progression and identifying the prognostic markers are important for treatment optimizing, patient care and long-term planning.

The mini-mental state examination (MMSE) is a widely accepted measurement of global cognition. The reported cognitive deterioration per year varies from 2.7–4.5 points [5]. The Kungsholmen study reported −2.8 points in MMSE during the first 3 years and −3.0 in the following 4 years [6]. Another community-based study showed that the average annual decline of MMSE, Alzheimer’s disease Assessment Scale-Cognition (ADAS-cog) and Disability Assessment for dementia (DAD) were 2.3, 11.4 and 15.1 points, respectively [7]. However, although being useful in screening the cognitive impairment, MMSE has limited value in measuring the progression of dementia because of substantial variation among individuals. [8] Domain-specific cognitive measure showed that in the presymptomatic stage of AD, memory and executive function showed the greatest decline and indicate fast progression. [9], [10] However, few studies reported the cognitive deterioration pattern in the clinical dementia stage. With regard to the mandarin-speaking Chinese, studies with whole neuropsychological battery across the cognitive spectrum were even more limited.

As to the risk factors of fast decline, many predictors of faster decline have been studied, such as early age at onset (AAO), higher education, family history, less leisure activities, psychotic symptoms, functional disability, and apolipoprotein E ε4 allele [11]–[14], but inconsistent results have been obtained. Roselli found that high educations is associated with fast progression [15] while Pavlik and Mangone reported the opposite result [16], [17]. The role of comorbidity is also controversial. Diabetes is commonly known to modulate AD risk, but their influence on cognition decline is contradictory. Roselli stated that diabetes associated with fast decline [15] while another study found diabetes to be a protective factor for disease progression [18]. These inconsistencies may be attributable to different study samples (size, patient selection), length of follow-up, statistical methodology (linear vs. non-linear decline) and interactions among the investigated variables.

In the present study, we followed clinically diagnosed AD patients from the memory clinic in Shanghai, China, and administered the MMSE and a battery of other neuropsychological tests to assess the rate of cognitive decline and to analyze the related factors.

## Methods

### Participants

Patients were recruited from the memory clinic of Huashan Hospital between January 1 2003 and December 31 2006. All patients had been clinically diagnosed with AD according to the National Institute of Neurological and Communicative Disorders and Stroke/Alzheimer’s Disease and Related Disorders Association criteria (NINCDS-ADRDA) [19]. Dementia or cognitive impairment due to other disorders such as vascular disease, Lewy body disease, progressive supranuclear palsy, and normal pressure hydrocephalus, etc were excluded. The study was approved by the Independent Review Board of Huashan Hospital. Written informed consent was obtained from participants and their proxies.

A total of 608 probable AD patients were enrolled and accepted at baseline examination. Among these, 134 were lost during follow-up. Of the 474 remaining patients, 165 were followed-up clinically in a face-to-face manner and neuropsychological assessments were administered, whereas 309 only accepted telephone follow-up to provide information on survival and complications. Neuropsychological test results and other information for the 165 patients were analyzed for cognitive changes and disease progression.

### Clinical Evaluation

Each participant underwent a semi-structured evaluation at baseline, including data on age, gender, educational status, and medical history (hypertension, diabetes, hyperlipidemia, heart disease, cerebrovascular disease, falls, and arthritis). Age at onset, diagnosis, clinical type (early or late onset), and family history were also recorded. Heart disease was defined as a history of congestive heart failure, myocardial infarction, or angina pectoris before the diagnosis of AD. Data were also collected regarding any treatment for hypertension, diabetes, hyperlipidemia, cerebrovascular diseases, falls, and arthritis at any time before the diagnosis of AD. Clinical types of early- or late-onset AD were defined as onset age<or ≥65 years old, respectively. Education was recorded as the number of years of formal education. All data were based on self-reports and/or medical records at baseline and follow-up visits. Self-reported information was confirmed by medical records for all cases whose medical records were available. The medical conditions were diagnosed on the basis of the details obtained from the patients or their attendants, if necessary.

Details regarding comorbid conditions and complications, including aspiration pneumonia, urinary tract infections, heart failure, cancer, cerebrovascular accident, decubitus ulcers, musculoskeletal disease, and metabolic disorders were obtained at follow-up. Musculoskeletal disease referred to all diseases involving the muscles, bones and skeleton. Metabolic disorder referred to any medical condition that interfered with metabolic system and function. Diabetes and gout were the commonest co-morbid diseases in this category. Arthritis was defined as any kind of arthritis. The interviewers, who were doctors, tried to establish the exact time when these medical conditions appeared. If the medical condition developed prior to the dementia symptoms, it was considered to be a co-morbid disease. If the medical condition developed after the dementia symptoms and diagnosis, interviewers tried to get more detailed information from the proxy. Group discussion involving the senior specialist was then held to decide if the condition should be classified as a complication or a co-morbid disease, on a case-by-case basis.

### Cognition Assessment

Global cognition was assessed using the MMSE. We administered Chinese version of Mini-Mental State Examination (C-MMSE, Zhang MY, 1989), which was mainly translated from the original one by Folstein and widely used as cognitive screening test in China. Although modified a little according to China’s cultural and economic situation, the scoring of C-MMSE was the same and its result was comparable to the original one. It is now widely accepted to set different cutoff points for MMSE score according to the respondents’ level of education. Practically, we adopted the C-MMSE cutoff points as following: 19 for the illiterate, 22 for those who had received less than six years education and 26 for those who had received at least six years education.

In addition, a complete neuropsychological battery including the following tests was administered to patients with mild to moderate AD at baseline and during follow-up:

1. Auditory verbal learning test [20], [21] (AVLT), which evaluates a wide range of auditory and verbal learning functions. Immediate recall, short and long delayed recall were recorded (AVLT_short delayed recall_ and AVLT_long delayed recall_).
2. Logic memory test [21], [22] (LM) assesses immediate recall (LM_immediate recall_) and delayed recall (LM_delayed recall_) of a short story.
3. Stroop color-word test[23]–[25] (Stroop) assesses the time taken to correctly identify the number of instances in which the color of a written word is identified (A_time_ and A_correct_), color of symbol (B_time_ and B_correct_) and the color of the ink rather than the color the word spells (C_time_ and C_correct_).
4. Complex figure test [21], [26] (CFT) has two components and measures both visuo-spatial constructional ability (CFT_copy_) and visuospatial memory (CFT_recall_).
5. Verbal fluency test [27] (VFT) assesses the correct number of animal, vegetable, fruit, and common grocery items identified within 1 min (VFT_animal_, VFT_vegetable_, VFT_fruit_, VFT_grocery_).
6. Trail making test [28] (TMT): TMTA and TMTB indicate the mean times taken to complete part A (visual conceptual, TMT_A) and part B (visuo-motor tracking, which involves motor speed and attention functions, TMT_B).
7. Huashan naming test [29] (HNT): a 100-item version of the HNT was administered, and the number of correct names was recorded.
8. Clock drawing test [30] (CDT): the participants were asked to draw the face of a clock. A 30-point scoring system was adopted.
9. Five-point test [31] (FPT): one of the various measures of figural fluency functions as nonverbal analogues to word fluency tasks, to evaluate the ability to initiate and sustain mental productivity in the visual-spatial domain. The number of correct answers was recorded.

All these neuropsychological tests have been validated for use in the Chinese population [20]–[22], [25]–[28], [32].

Five cognitive domains were assessed based on the above-mentioned tests: (1) memory: AVLT, LM; (2) visuo-spatial ability: CFT, CDT, FPT; (3) language: VFT, HNT; (4) executive function: Stroop_C, TMT_B; (5) attention: Stroop_A, TMT_A. The clinical dementia rating scale (CDR) was also applied.

### Data Analysis

All cases that satisfied the inclusion criteria and finished the follow-up cognition assessment were included in the analysis. The characteristics of subjects and related information at diagnosis were analyzed. Change patterns in memory and cognition over time were evaluated using paired t-tests for each neuropsychological test.

Because each neuropsychological test yielded an individual test score that could not be compared directly with each other, and because of “ceiling/floor effects” in the raw scores, composite indexes were used for each cognitive domain and global cognition. Each raw score was transformed into a standardized Z score based on its mean and standard deviation (SD) calculated from the cognitively normal population[20], [22], [25]–[28], [32], according to the formula: Z = (raw score – mean score)/SD [12], [33]–[35]. Individual cognitive measures were grouped into specific cognitive domains. Within each domain, z scores were averaged to yield composite scores that were used in second analysis. Memory: AVLT_short delayed recall_, AVLT_long delayed recall_, LM_immediate recall_, LM_delayed recall_, CFT_recall_; Visuo-spatial: CFT_copy_, CDT, FPT; Language: VFT, HNT; Executive: Stroop_C_Cr_, TMT_B; and Attention: Stroop _A_ Cr_, TMT_A. The composite global Z score was the average of the five domain scores, with missing data treated as described.

Linear regression analysis was performed to identify any factors or predictors associated with the deterioration of cognition. Univariate and multivariate regression were used. Covariates were chosen according to 1) any factor with a p value ≤0.2 in univariate analysis; 2) covariates known to be associated with AD incidence or progression based on the literature. The significant level was set at 0.05 for P value. Pearson’s correlation analysis was conducted to explore the association among the cognition domains.

## Results

### 1. Descriptive Statistics

The participants were the clinically diagnosed probable AD patients from the memory clinic at Huashan Hospital, Fudan University, Shanghai, China. A total of 165 patients received face-to-face follow-up and were enrolled in the analysis of cognitive changes. There were 57 males and 108 females, with a mean age of 74.19±8.83 years and an average of 8.55±4.97 years’ education. The baseline MMSE score was 15.52±5.86. The time interval between baseline and follow-up visits ranged from 1 to 5.28 years (2.57±0.99).

### 2. Cognitive Changes Over Time

Participants’ cognitive function was reevaluated by repeating the neuropsychological tests performed at baseline. MMSE was administered to all 165 patients at both baseline and follow-up visits. Patients with mild to moderate AD received other neuropsychological tests. The annual change in each raw score was calculated using (follow up – baseline)/years followed. All the participants came from the memory clinic of Huashan Hospital. Once they were diagnosed Alzheimer’s disease, they received anti-dementia medication, such as AchEIs and memantine, etc. In this regard, they were all AD patients under formal treatment. The MMSE score declined at a rate of 1.52 per year. A “floor effect” was observed in AVLT delayed recall, LM and CFT recall; subjects had very poor scores (almost zero) at baseline, thus no significant difference was detected at follow-up. The scores of all the other neuropsychological tests deteriorated significantly over time. Attention (−156.04%) and executive function (−59.18%) showed much more rapid rates of decline than those for memory and language (0.32% and −1.54%, respectively). The composite Z scores for the five cognitive domains were shown in Table 1.

**Table 1:** Neuropsychological Raw and Z scores at Baseline and Follow-up Visits.

	No	Baseline	Follow-Up	Annual change (%)
MMSE	165	15.52±5.86	11.76±7.02**	−1.52±2.29 (−9.79%)
AVLT_short delayed recall_	45	0.00±0.00	–	–
AVLT_long delayed recall_	44	0.05±0.21	–	–
LM_immediate recall_	50	2.36±1.96	1.60±2.04*	−0.20±1.32 (−8.47%)
LM_delayed recall_	49	0.29±0.98	–	−
Stroop _A_ Cr_	49	48.92±1.80	41.18±17.21**	−3.66±8.64 (−7.48%)
Stroop _B_Cr_	49	44.63±4.80	33.82±17.92**	−5.16±8.72 (−11.56%)
Stroop_C_Cr_	49	29.61±11.38	19.71±16.89**	−4.29±6.55 (−14.49%)
CFT_copy_	43	23.02±12.58	15.91±14.34**	−3.59±5.14 (−15.60%)
CFT_recall_	43	2.63±10.48	1.37±4.32	−0.04±2.90 (−1.52%)
VFT_animal_	48	9.38±3.34	7.38±3.89**	−0.74±2.16 (−7.89%)
VFT_vegetable_	45	6.69±2.29	4.91±3.12**	−0.69±1.61 (−10.31%)
VFT_fruit_	45	7.02±3.25	5.36±3.56**	−0.71±1.75 (−10.11%)
VFT_grocery_	66	8.86±4.49	7.35±5.24**	−0.66±2.23 (−7.45%)
TMT_A	22	94.36±46.04	128.27±63.42**	12.78±28.05 (13.54%)
TMT_B	15	240.87±96.69	318.00±107.79*	25.72±60.22 (10.68%)
CDT	45	13.42±8.32	9.73±9.31**	−1.48±3.96 (−11.03%)
FPT	22	2.68±2.28	2.59±2.96	−0.04±2.26 (−1.49%)
HNT	28	73.82±18.31	66.18±22.59**	−4.51±7.77 (−6.11%)
CDR	147	1.88±0.79	2.49±1.06	0.23±0.35(12.23%)
Z_total_	100	−1.87±1.18	−2.99±2.16	−0.53±0.79 (−28.34%)
Z_mem_	55	−3.10±0.43	−3.14±0.47	0.01±0.21 (0.32%)
Z_exe_	49	−2.45±1.70	−5.50±3.68	−1.45±1.81 (−59.18%)
Z_visuo_	49	−3.59±3.54	−5.79±4.45	−1.08±1.69 (−30.08)
Z_lang_	94	−1.30±0.95	−1.73±0.90	−0.20±0.36 (−1.54%)
Z_att_	49	−0.91±1.15	−3.94±5.16	−1.42±2.41 (−156.04%)

Mean±SD; FU(follow-up) vs baseline, *p<0.05, **p<0.01; Annual change = (FU-baseline)/years followed. Annual change% = (Annual change/baseline score)*100%.

MMSE: mini-mental status examination; AVLT: auditory verbal learning test; LM: logic memory test; Stroop: Stroop color word test; CFT: complex figure test; VFT: verbal fluency test; TMT: trail making test; CDT: clock drawing test; FPT: five point test; HNT: Huashan naming test. Z_mem_: Z score of memory; Z_exe_: Z score of executive function; Z_visuo_: Z score of visuo-spatial function; Z_lang_: Z score of language; Z_att_: Z score of attention function; Z_total_: Z score of global cognition.

### 3. Factors Associated with Cognitive Decline

Analysis was performed to identify any factors or predictors associated with cognitive deterioration. There were no differences between the unadjusted models and those adjusted by covariates. Here only the adjusted models are presented. No factor was significantly associated with the change in MMSE score. Composite Z scores for each cognitive domain and general cognition were calculated as described above. Multivariate regression analysis identified age and age at onset to be associated with global cognition change. Younger patients and early-onset AD patients deteriorated more rapidly than older and late-onset patients (Table 2). In terms of Z score for each cognition domain, men declined faster in memory but slower in attention than women. In executive and attention functions, the rates of decline of these two cognitive domains differed greatly between early- and late-onset patients and in different age groups. Highly educated patients deteriorated more rapidly in visuo-spatial ability.

**Table 2:** Deterioration in Measures of Cognition for Various Risk Factors in Alzheimer’s Disease (Annual change).

Riskfactors		No	Value	MMSE	Z_total_	Z_mem_	Z_exe_	Z_visuo_	Z_lang_	Z_att_
	**Overall** **change**	165	Mean(range)	−1.52(−9.76∼5.01)	−0.32(−3.02∼−1.08)	0.10(−1.47∼3.07)	−0.36(−2.06∼0.89)	−0.18(−1.35∼0.87)	−0.21(−1.25∼1.08)	−1.26(−13.63∼26)
**Gender**	M	57	Mean(CI)	−1.67(−2.35∼−0.98)	−0.25(–0.37∼−0.13)	**0.01** **(**−**0.09∼0.10)^$^**	−0.26(−0.44∼−0.08)	−0.18(−0.35∼−0.01)	−0.21(−0.32∼−0.10)	−**0.65** **(**−**1.26∼**−**0.03)^$^**
	F	107	Mean(CI)	−1.45(−1.86∼−1.04)	−0.36(−0.55∼−0.18)	0.19(−0.11∼0.49)	−0.45(−0.72∼−0.18)	−0.18(−0.38∼−0.02)	−0.22(−0.34∼−0.10)	−1.79(−3.07∼−0.52)
**Age**	<65	27	Mean(CI)	−1.99(−2.89∼−1.08)	−**0.79** **(**−**1.20∼**−**0.19)** **	0.03(−0.30∼0.36)	−**0.71** **(**−**1.10∼**−**0.33)** ** **^$^**	−0.28(−0.58∼0.02)	−0.23(−0.43∼−0.03)	−**3.03** **(**−**5.38∼**−**0.69)** **
	≥65	137	Mean(CI)	−1.43(−1.82∼−1.05)	−0.23(−0.32∼−0.14)	0.13(−0.06∼0.32)	−0.21(−0.36∼−0.06)	−0.15(−0.29∼−0.01)	−0.21(−0.30∼−0.12)	−0.54(−0.88∼−0.21)
**Education**	<6	40	Mean(CI)	−1.48(−2.30∼−0.67)	−0.26(−0.46∼−0.06)	0.17(−0.37∼0.70)	−0.01(−0.71∼0.70)	**0.18** **(**−**0.56∼.92)^$^**	−0.28(−0.47∼−0.10)	−0.09(−0.44∼0.25)
	≥6	122	Mean(CI)	−1.56(−1.96∼−1.16)	−0.32(–0.45∼−0.19)	0.10(−0.08∼0.27)	−0.40(−0.57∼−0.23)	−0.22(−0.35∼−0.10)	−0.20(−0.29∼−0.11)	−1.39(−2.19−0.58)
**Age at** **onset**	Early	37	Mean(CI)	−1.71(−2.39∼−1.02)	−**0.52** **(**−**0.89∼**−**0.15)** * **^$^**	0.06(−0.24∼0.35)	−**0.54** **(**−**0.92∼**−**0.16)^$^**	−0.20(–0.48∼0.08)	−0.21(−0.36∼−0.06)	−**2.58** **(**−**4.70∼**−**0.47)** ** **^$^**
	Late	126	Mean(CI)	−1.51(−1.92∼−1.10)	−0.24(−0.33∼−0.15)	0.12(−0.77∼0.32)	−0.26(−0.42∼−0.11)	−0.17(−0.32∼−0.03)	−0.22(−0.31∼−0.12)	−0.61(−0.96∼−0.26)
**BMI**	<24	104	Mean(CI)	−1.56(−2.02∼−1.10)	−0.37(−0.53∼−0.21)	0.13(−0.08∼0.35)	−0.34(−0.55∼−0.14)	−0.18(−0.38∼0.02)	−0.25(−0.36∼−0.15)	−1.66(−2.81∼−0.52)
	≥24	47	Mean(CI)	−1.59(−2.28∼−0.90)	−0.25(−0.41∼−0.09)	0.08(−0.21∼0.37)	−0.47(−0.73∼−0.21)	−0.19(−0.33∼−0.03)	−0.20(−0.32∼−0.07)	−0.67(−1.29∼−0.04)
**HBp**	Y	64	Mean(CI)	−1.40(−1.95∼−0.86)	−0.22(−0.36∼−0.09)	0.15(−0.17∼0.47)	−**0.26** **(**−**0.45∼**−**0.07)^$^**	−0.16(−0.30∼−0.02)	−0.18(−0.28∼−0.09)	−0.83(−1.48∼−0.19)
	N	98	Mean(CI)	−1.64(−2.11∼−1.16)	−0.38(−0.56∼−0.21)	0.05(−0.04∼0.15)	−0.46(−0.72∼−0.19)	−0.20(−0.44∼0.03)	−0.24(−0.36∼−0.12)	−1.70(−3.06∼−0.33)
**CHD**	Y	41	Mean(CI)	−1.06(−1.63∼−0.50) *	−0.19(−0.38∼−0.06)	0.20(−0.27∼0.66)	−0.18(−0.52∼0.15)	−0.02(−0.27∼0.22)	−0.17(−0.32∼−0.03)	−0.59(−1.30∼0.12)
	N	117	Mean(CI)	−1.79(−2.23∼−1.36)*	−0.36(−0.50∼−0.22)	0.06(−0.06∼0.18)	−0.43(−0.61∼−0.24)	−0.25(−0.40∼−0.10)	−0.23(−0.32∼−0.13)	−1.55(−2.56∼−0.54)
**CVD**	Y	35	Mean(CI)	−1.37(−2.31∼−0.44)*	−0.17(−0.33∼−0.01)	**0.44** **(**−**0.21∼1.09)** * **^$^**	−0.15(−0.36∼0.07)	−0.01(−0.31∼0.28)	−0.19(−0.33∼−0.06)	−0.78(−1.61∼0.06)
	N	122	Mean(CI)	−1.66(−2.05∼−1.28)	−0.35(−0.49∼−0.21)	0.01(−0.10∼0.11)	−0.42(−0.62∼−0.22)	−0.23(−0.37∼−0.09)	−0.22(−0.32∼−0.12)	−1.40(−2.32∼−0.47)
**DM**	Y	21	Mean(CI)	−1.12(−1.96∼−0.28)	−0.23(−0.49∼−0.02)	0.15(−0.04∼0.34)	−0.13(−0.68∼0.41)	−0.14(−0.49∼0.21)	−0.10(−0.26∼0.07)	−1.14(−2.73∼0.44)
	N	141	Mean(CI)	−1.61(−2.00∼−1.22)	−0.33(−0.45∼−0.20)	−0.11(−0.25∼0.03)	−0.40(−0.57∼−0.22)	−0.19(−0.33∼−0.05)	−0.24(−0.32∼−0.15)	−1.27(−2.11∼−0.44)
**Smoke**	Y	15	Mean(CI)	−1.86(−3.02∼−0.51)	−0.30(−0.62∼−0.03)	0.10(−0.24∼0.45)	−0.10(−0.55∼0.36)	−0.15(−0.72∼0.42)	−0.30(−0.59∼−0.0)	−0.99(−2.77∼0.78)
	N	142	Mean(CI)	−1.58(−1.95∼−1.20)	−0.31(−0.44∼−0.19)	0.10(−0.08∼0.28)	−0.40(−0.57∼−0.22)	−0.19(−0.31∼−0.06)	−0.20(−0.29∼−0.12)	−1.29(−2.11∼−0.48)
**Alcohol Drinking**	Y	15	Mean(CI)	−1.68(−3.30∼−0.07)	−0.20(−0.60∼0.20)	0.22(−0.54∼0.98)	0.09(−1.35∼1.53)	−0.05(−1.35∼1.25)	−0.29(−0.72∼0.14)	−0.28(−0.84∼0.29)
	N	146	Mean(CI)	−1.53(−1.90∼−1.16)	−0.32(−0.44∼−0.20)	0.09(−0.08∼0.26)	−0.39(−0.55∼−0.22)	−0.19(−0.31∼−0.07)	−0.21(−0.29∼−0.13)	−1.32(−2.09∼−0.54)
**Family history**	Y	36	Mean(CI)	−1.47(−2.12∼−0.82)	−0.36(−0.61∼−0.11)	−0.10(−0.35∼0.16)	−**0.61** **(**−**1.09∼**−**0.13)^$^**	−0.38(−0.58∼−0.17)	−0.25(−0.42∼−0.07)	−1.42(−3.15∼0.32)
	N	118	Mean(CI)	−1.62(−2.05∼−1.18)	−0.32(−0.45∼−0.19)	0.17(−0.03∼0.37)	−0.29(−0.45∼−0.12)	−0.11(−0.27∼0.04)	−0.23(−0.31∼−0.14)	−1.24(−2.10∼−0.38)
**Comorbid/** **complication**	Y	85	Mean(CI)	−1.67(−2.16∼−1.18)	–0.31(−0.48∼−0.14)	0.19(−0.08∼0.46)	−0.34(−0.53∼−0.15)	−0.16(−0.34∼0.03)	−0.25(−0.35∼−0.14)	−1.32(−2.62∼−0.02)
	N	73	Mean(CI)	−1.53(−2.07∼−1.00)	−0.31(−0.47∼−0.16)	0.01(−0.16∼0.18)	−0.38(−0.64∼−0.11)	−0.21(−0.39∼−0.03)	−0.18(−0.30∼−0.07)	−1.20(−2.05∼−0.35)

Z_mem_: Z score of memory; Z_exe_: Z score of executive function; Z_visuo_: Z score of visuo-spatial function; Z_lang_: Z score of language; Z_att_: Z score of attention function; Z_total_: Z score of global cognition; BMI: body mass index; HBp: hypertension; CHD: coronary heart disease; CVD: cerebrovascular disease; DM: diabetes.

Univariate analysis:

*p<0.05,

**p<0.01;

Multivariate analysis: ^$^p<0.05, ^$$^p<0.01; Annual change = (follow up-baseline)/years followed.

### 4. Correlations among Cognitive Domains

Pearson’s bivariate correlations were performed among MMSE and all the composite Z scores. MMSE correlated well with each Z score. Executive and visuo-spatial Z score closely correlated with all the other Z scores (Table 3), implying that these two cognitive functions relied on all the other cognitive domains. Each cognitive domain correlated well with MMSE, and contributed to global cognition. Executive function correlated with the other four cognitive domains.

**Table 3:** Correlation of Annual Decline in Each Cognitive Domain.

	MMSE	Z_mem_	Z_exe_	Z_visuo_	Z_lang_	Z_att_	Z_total_
**MMSE**	1(164)	0.335* (54)	0.426** (48)	0.637** (49)	0.352** (94)	0.295* (49)	0.401** (96)
**Z_mem_**	**–**	1(54)	0.209(48)	0.407** (49)	0.252(52)	0.122(49)	0.375** (54)
**Z_exe_**	**–**	**–**	1(48)	0.497** (43)	0.604**(47)	0.494** (48)	0.700** (48)
**Z_visuo_**	**–**	**–**	**–**	1(49)	0.549**(47)	0.065(44)	0.422** (49)
**Z_lang_**	**–**	**–**	**–**	**–**	1(94)	0.273(48)	0.658** (94)
**Z_att_**	**–**	**–**	**–**	**–**	**–**	1(49)	0.916** (49)

*p<0.05,

**p<0.01.

Z_mem_: Z score of memory; Z_exe_: Z score of executive function; Z_visuo_: Z score of visuo-spatial function; Z_lang_: Z score of language; Z_att_: Z score of attention function; Z_total_: Z score of global cognition.

## Discussion

Almost all the cognitive domains memory declined significantly over time, but at different rates. Attention, executive and visuo-spatial functions declined more aggressively than other cognition domains (memory and language). Studies had shown that, from mild cognitive impairment (MCI) to the early stage of AD, memory was the earliest and main affected domain [9]. When the disease progressed to the moderate to severe stage, all other cognitive fields were affected without exception. Attention, executive and visuo-spatial functions would eventually be affected, while language would be the last cognitive domain to be affected. Understanding the sequence of cognitive impairment in each domain in AD patients may help neurologists and caregivers to manage the disease better, as well as providing more information for treatment. Zhou [36] reported that a good baseline performance in executive function predicted longer survival in AD patients, which suggests that more attention should be paid in clinical practice to AD patients’ executive function. Our study failed to find significant change in memory. This is because memory tests here, such as AVLT, are not suitable for assessing disease progression in moderate to severe AD (“floor effect”). The patients in our study were relatively severe, as shown that the mean baseline MMSE score is 15.52. The mean delayed recall score at baseline is near zero. Thus, no change could be observed during the follow-up.

Factor analysis revealed that cognition declined faster in early-onset AD (EOAD) patients than in late-onset AD (LOAD). Men declined faster than women in terms of memory, but slower than women in attention function. Family history was associated with worsening of executive function. In addition, more-educated patients deteriorated faster in terms of visuo-spatial ability than less-educated ones.

The finding of a poorer prognosis in younger and early-onset patients is not unique. Previous studies have identified similar trends of rapid declines in such patients [11], [37], [38]. Bernick also reported that in AD clinical trials, older age was associated with a slower rate of decline in the ADAS-cog and the MMSE. [39] AD is an age-related disorder in which the pathologic changes are thought to be present decades before symptom appears. Younger patients have generally better reserves both in physical status and cognition. Therefore, when dementia is clinically manifested in younger subjects, it is reasonable to expect that more advanced pathological change is present in the brain which indicates more advanced stage and eventually more aggressive progress [11].

The relationship between educational status and the rate of cognitive decline has been controversial [14], [40], [41]. Our results indicated a slightly faster decline in visuo-spatial ability in more-educated patients. Some but not all epidemiological studies had also noted faster progression in high-educated AD. This was attributed to habouring a higher pathological burden at the time of clinical dementia. Wilson hypothesized that education might diminish the effects of AD pathology on cognition [14]. Once dementia is clinically manifested, however, more AD-like pathology may accumulate in those with higher educational attainment, eventually resulting in a more rapid decline during the later stages of the disease process [14]. Our observation is consistent with this hypothesis.

Regarding to the gender differences, we only discussed the attention function here since memory alteration was biased by the very low baseline scores in related tests (“floor effect”). “Attention” reflects the speed of processing and conceptual abilities [24]. Our results suggest that Chinese women perform worse and decline faster than men in “attention” abilities. This may attribute to differences in life experiences between the two genders in China.

Many studies have reported an association between vascular factors and AD progression. Mielke found hypertension associate with rapid decline in MMSE and CDR, while diabetes associate with slower decline [42]. Sanz and Musicco studied a cohort of AD patients and found that diabetes was associated with a slower rate of cognitive decline [11], [18]. Regan et al. proposed that vascular risk factors may contribute to the initial expression of AD, but may not involve in the underlying etiologic process [43]. Our results suggested that hypertension was associated with a slower rate of executive function decline, whereas cerebrovascular disease with slower declines in memory. The explanation was that AD patients with vascular risk factors was not “pure”. The underlying etiology involved both degenerative and vascular pathologies. The vascular risk factors or underlying vascular lesions were modified or reversed by medications or lifestyle change, resulting in fluctuations in cognitive function over time. In addition, the biased survival of cerebrovascular disease also accounted at least partly for the slower decline. Further studies are needed to clarify this observation before recommendations can be made.

There were several advantages of this study. It was a clinical-based survey that could provide useful information on disease progression profiles. Patients were assessed using not only MMSE test, but also a battery covering memory, executive, attention, visuo-spatial functions and language, allowing analysis for cognitive components.

Despite these strengths, several limitations warrant consideration. This was a retrospective study. Although we tried to contact all the patients, some were lost to follow-up. A preliminary analysis showed that the clinically-followed participants were younger, better educated, and had higher baseline MMSE scores than those could not be followed (data not shown). The lost cases may have already deceased or progressed to the advanced stages. Therefore, our results were biased to slower progression. It is possible that the analysis may lack the power to detect risk factors associated with disease progression. Continuation of the survey, with the involvement of more patients and longer-term follow-up, will provide more accurate data and clarify these results. In addition, neuropsychological tests for evaluating disease progression, especially memory tests, need to be carefully selected to avoid the “floor effect”. It is possible that some risk factors associated with memory decline were missed for this reason. Another important limitation of the study is that the follow-up length is heterogeneous; the annual change of the neuropsychological index is only a crude picture of the AD cognitive decline. We also acknowledged that in different stages of the disease, the cognition decline may have unique pattern, but in this study, the limited sample size prohibited us to do further sub-stage analysis. Prospective follow-up study may provide us the different cognition changing pattern overtime. Thus, further prospective study with strict routine follow-up is urgently needed.

## Conclusions

The results of this retrospective hospital-based study suggest that almost all the cognitive domains decline over time. Attention, executive, and visuo-spatial functions deteriorating at a faster rate. Age, age at onset, and educational status were associated with disease progression. Further studies are needed to confirm these findings and to determine the mechanisms behind these associations.
